# Identification and characterization of N-glycosylation site on a *Mucor circinelloides* aspartic protease expressed in *Pichia pastoris*: effect on secretion, activity and thermo-stability

**DOI:** 10.1186/s13568-018-0691-3

**Published:** 2018-10-01

**Authors:** Martin Kangwa, Jose Antonio Gama Salgado, Hector Marcelo Fernandez-Lahore

**Affiliations:** Downstream Bioprocessing Laboratory, Department of Life Sciences & Chemistry, Jacobs University, Campus Ring 1, 28759 Bremen, Germany

**Keywords:** Aspartic proteinase, *Mucor circinelloides*, Secretion signal peptide, *Pichia pastoris*, milk-clotting unit (MCU), N-glycosylation

## Abstract

Methylotrophic yeasts have widely been used as model organisms for understanding cellular functions and biochemical activities in lower eukaryotes. The gene encoding an aspartic protease (MCAP) from *Mucor circinelloides* DSM 2183 was cloned and expressed into *Pichia pastoris* using both the native *M. circinelloides* signal peptide (mcSP) and α-factor secretion signal from *Saccharomyces cerevisiae* (α-MF). When expressed in *P. pastoris* using α-MF and mcSP, MCAP was secreted into the culture medium at a concentration 200 mg L^−1^ (410 MCU mL^−1^) and 110 mg L^−1^ (249 MCU mL^−1^), respectively. The SDS-PAGE analysis of each culture shows that the protein was secreted in the media in two forms with molecular weights of approximately 33 and 37 kDa. Upon digestion using endoglycosidase H (Endo H), only one band at 33 kDa was observed, indicating that the protein might be glycosylated. One putative N-glycosylation site was found and a site-directed mutagenesis at position Asn331-Gln of the sequence produce only one form of the protein of 33 kDa, similar to that obtained when digested with Endo H. The optimum temperature and pH activity of the expressed MCAP was found to be at 60 °C and 3.6, respectively.

**Electronic supplementary material:**

The online version of this article (10.1186/s13568-018-0691-3) contains supplementary material, which is available to authorized users.

## Introduction

Methylotrophic yeast, mainly *Pichia pastoris* have emerged as an attractive industrial strain for the production of viable proteins (Yang et al. 2015; Daly and Hearn 2005), as they are considered to possess several advantages, including low cost and large-scale production, easy to scale-up, harvesting, and storage. They are able to produce high yields of secreted biologically active valuable recombinant proteins (Daly and Hearn 2005; Dietzsch et al. 2011). One of the principal advantages of yeast production systems is their ability to perform the post-translational modification, which is required for glycosylation, phosphorylation, and methylation, as well as proteolytic processing and non-enzymatic modifications for biological activity and stability (Spadiut and Herwig 2014; Dietzsch et al. 2011; Walsh and Jefferis 2006).

The attachment of sugar molecules to protein is one of the most well-known and vital forms of co- and post-translational modification as the addition can significantly change the structure, and subsequently the function of the protein. The addition of sugar residues can occur at certain amino acid side-chains, and these modifications can be *N*- and *O*-glycosylation type, phosphoglycation, C-mannosylation, and glypiation (Fitchette-Lainé et al. 1998; Spiro 2002). *P. pastoris* is known to N-glycosylate proteins via mannose oligosaccharide linked to asparagine via its two N-acetylglucosamines and these diverse roles of glycans can affect the conformational maturation, secretion, activity, and stability of glycoproteins (Laukens et al. 2015; Yang et al. 2015). This modification can either be advantageous or not depending on the application field of the desired protein. Detailed studies on deglycosylation conducted on aspartic proteinase of *Mucor pusillus* have revealed that the elimination of N-linked carbohydrate groups from the protein affects the enzyme properties such as milk-clotting and proteolytic activities while leading to a decrease in the level of secretion and thermal stability in other fungi (Aikawa et al. 1990; Yamashita et al. 1994). Moreover, a high degree of glycosylation has been proposed to be the purpose for a well-stabilized structural conformation, which may confer a high level of thermal stability for the aspartic proteinase from *Rhizomucor miehei* (Celebi et al. 2016; Soltani et al. 2016)*, M. pusillus, Sporisorium reilianum* and *A. niger* (Mandujano-González et al. 2016; Rickert and McBride-Warren 1977; Zhang et al. 2015).

Aspartic proteinases (APs; EC.3.4.23.-) also known as acid proteases are a significant group of enzymes, produced by a variety of organisms including plants and animals, mainly used as milk coagulating agents in industrial cheese production (Yegin and Dekker 2013; Hsiao et al. 2014). Rennet, a milk coagulant obtained from the abomasum of milk-fed lambs and calves, is regarded as the industrial gold standard in cheese manufacturing (Kethireddipalli and Hill 2015). Chymosin (EC 3.4.23.4) is the main milk-clotting component of natural rennet with activity amounting to about 90% of the total observed potency (Salehi et al. 2017; Kumar et al. 2010). Chymosin specifically cleaves the Phe_105_-Met_106_ bond of κ-casein (κ –CN) resulting in the formation of the (hydrophobic) para-κ-casein and the (hydrophilic) biologically active component glycomacropeptide (Neelima et al. 2013). The glycomacropeptide further diffuses into cheese whey and the destabilized micelles consequently aggregate to form cheese curd via the Ca^2+^ induced aggregation (Hang et al. 2016; El-Baky et al. 2011). Due to increased demand for cheese products, animal-derived milk coagulants are not enough to cover the production, and this has prompted research efforts in the search, manufacture of recombinant and microbial rennin. Substitutes with milk coagulating ability must present some of calf rennet specific properties like a high ratio of milk clotting activity and suitable thermal stability. Microbial milk coagulants, also known as mucor rennins produced by microorganisms like *Rhizomucor miehei* (Gray et al. 1986; Ottesen and Rickert 1970), *Rhizomucor pusillus* (Arima et al. 1968; Tonouchi et al. 1986), and *Endothia parasitica* have found extensive application as milk coagulating enzymes in industrial cheese production (Jacob et al. 2011; Nasr et al. 2016). However, these fungal enzymes have several disadvantages when compared to their animal counterpart rennet, due to the flavour and bitter taste that dominate the cheese products; produced especially after a long period of maturation. For this reason, researchers have focused on finding new enzymes that possess the desired properties and characteristics dearly needed in cheese manufacturing. With this goal in focus, aspartic proteinase was isolated from *M. circinelloides* and further characterized.

In our previous studies, the aspartic protease gene from *M. circinelloides* (GenBank accession no. JQ906105 and JQ906106) was cloned and expressed in *P. pastoris* (Gama Salgado et al. 2013). In this, present work, an *M. circinelloides* aspartic proteinase (MCAP), GenBank accession no. JQ906105 and JQ906106 with its native signal peptide (mcSP) were successfully expressed in *P. pastoris*. We further focused on identifying and analyzing the N-glycosylation site of an *M. circinelloides* aspartic protease and analyzing its effect on secretion, activity, and thermostability.

## Materials and methods

### Fungal, bacterial strains, and media

*Escherichia coli* Top10 strain (Invitrogen, Darmstadt, Germany), (Table 1) was used for all cloning procedures and grown with shaking at 220 rpm at 37 °C in Luria–Bertani medium (5 g L^−1^ yeast extract, 10 g L^−1^ tryptone, 5 g L^−1^ NaCl). Recombinant *P. pastoris*, was screened on YPDS agar plates (20 g L^−1^ peptone, 10 g L^−1^ yeast extract, 40 g L^−1^ glucose, 1 M sorbitol and 2% agar) containing 100 µg mL^−1^ of Zeocin (Invitrogen, Darmstadt, Germany). *Pichia pastoris* X-33 and SMD1168 strains (Invitrogen, Darmstadt, Germany), were used as platform strains for all engineered *P. pastoris*, and were grown at 24 °C for 72 h in 250 mL shake flask containing 75 mL of YPD liquid media (20 g L^−1^ peptone, 10 g L^−1^ yeast extract, 40 g L^−1^ glucose, pH: 5.0 adjusted with citric acid) in a shaking incubator at 250 rpm. All components for media preparation were purchased from Carl Roth GmbH, Germany. Media, electro-competent *E.coli* and *P. pastoris* cells were prepared according to the Expression kit manual (Invitrogen).

**Table 1:** Microorganisms and plasmids used in this study

Strain or plasmid	Genotype	Source or references
*Escherichia coli*		
Top10	F- mcrA Δ(mrr-hsdRMS-mcrBC) φ80lacZΔM15 ΔlacX74 recA1 araD139 Δ(ara-leu) 7697 galU galK rpsL (StrR) endA1 nupG	Invitrogen
*Pichia pastoris*		
X-33	Wild type	Invitrogen
SMD1168	Protease deficient	Invitrogen
Plasmids		
pGAPZα-A	The pGAPZα-A vector uses the GAP promoter to constitutively express recombinant proteins in *Pichia pastoris*	Invitrogen
pGAM1	A pGAPZα-A derived vector where the α-MF is replaced by mcSP and uses the GAP promoter to constitutively express recombinant proteins in *Pichia pastoris*	This work
pGAPZα + MCAP-5	pGAPZα-A derivative carrying the *MCAP* gene without a signal sequence and without introns^a^	Gama Salgado et al. (2013)
pGAM1 + MCAP-3	pGAPZα-A + MCAP-3 derivative carrying the MCAP with mcSF without an intron^b^	This work
pGAM1 + MutMCAP-8	pGAPZα-A + MCAP-3 derivative is carrying the MCAP without an intron. The codon of the amino acid asparagine 331(AAC) was changed to Glutamine (CAA)^b^	This work (Single mutant Asn331-Gln)
pGAM1 + SyMCAP-6	pGAM1 + SyMCAP-6 derivative carrying the *MCAP* gene with native signal sequence and without intron. The original *MCAP* gene was adapted to the optimal codon usage of *P. pastoris*. The insert was cloned flush with the Kex2 cleavage site and in frame of the native signal sequence and in frame with the C-terminal polyhistidine tag into the *XhoI* and *NotI* site of the pGAM1^b^	Gama Salgado et al. (2013) and in this work

^a^The insert was cloned flush with the *Kex2* cleavage site and in a frame of the native signal sequence and in a frame with C-terminal polyhistidine tag into the *XhoI* and *NotI* site of the pGAM1

^b^The insert was cloned downstream of the GAP promoter and in a frame with the C-terminal polyhistidine tag into the *XhoI* and *NotI* site of the pGAM1

### DNA manipulations, primers, and DNA sequencing

Taq DNA polymerase, T4 DNA ligase and restriction enzymes were purchased from New England Biolabs (NEB), while *Pfu* Ultra High Fidelity DNA Polymerase was from Stratagene, Heidelberg, Germany. All enzymes were used as specified by the manufacturer, plasmids were extracted using a QIAprep Spin Miniprep Kit, while the PCR products were purified using the QIAquick PCR Purification Kit (QIAGEN, Hilden, Germany). The synthesis of primers and nucleotides sequencings used in this study was performed by Eurofins MWG Operon (Eurofins MWG Operon, Ebersberg, Germany).

The PCR protocol for the construction of plasmids and for the identification of recombinant strains was a follow; 1 × ThermoPol reaction buffer, 200 µmol µL^−1^ dNTPs, 2 pmol µL^−1^ of each primer (forward and reverse), 0.6–1 ng µL^−1^ plasmid DNA, 0.04 units µL^−1^ *Taq* DNA polymerase final concentration. PCR reactions were carried in a total volume of 50 µL using a Mastercycler ep gradient S (Eppendorf, Hamburg, Germany).

### Plasmids construction

The plasmids used in this study can be found in Table 1. Expression vector pGAM1 + MCAP-3, containing the *MCAP* coding sequence with its native signal peptide sequence, was constructed as follows: the gene encoding *M. circinelloides* aspartic proteinase with its native *mcSP* sequence was PCR amplified using primer sequences MCAP-Fw 5′-TAT CTC GAG AAA AGA ATG AAA TTC TCA TTA GTC TCT TCT TGT G-3′ and MCAP-Rv 5′-TGG CGG CCG CGA CAG ATT TGG CAA TTT GGA C-3′ having restriction site *Xho*I and *Not*I site, respectively, producing the insert product *mcSP*-*MCAP*. PCR amplification was done using the *Pfu* Ultra High Fidelity DNA Polymerase (Stratagene, Heidelberg, Germany) consisting of 36 cycles of 94 °C for 30 s, 60 °C for 40 s and 68 °C for 2 min, and followed by a final extension step at 68 °C for 10 min, producing PCR gene product of about 1211 bp. After PCR the product was purified and digested using the enzyme *Xho*I and *Not*I. The vector backbone was digested and later ligated with the digested *mcSP*-*MCAP* and the resulting plasmid was the designated pGAM1 + MCAP-3, with the 6xHis tag at the C- terminal and the *bleo* gene as a selectable marker.

### Site-directed mutagenesis on N-glycosylation site sequence

Mutations on the glycosylation site were generated by a point mutation using the plasmid pGAM1 + MCAP-3 as the template. The codon for N (*Asn*331) in the glycosylation sequon was replaced with Q (*Gln*) to eliminate the N-glycosylation site using 5′-AAG CCT CTT cag TTC ACC ATC AAC GGT-3′ as the sense oligonucleotides to generate the mutant plasmid pGAM1 + MutMCAP-8. The plasmid was transformed into *E. coli*. The presence of the mutation was verified by nucleotide sequencing.

### *E. coli* and *P. pastoris* transformation

The plasmids were transformed into *E. coli* TOP10 competent cells and the cells were screened on Luria–Bertani plates containing 40 µg mL^−1^ of zeocin. Plasmids were isolated from overnight cultures of positive colonies and were sequenced for the insert of interest to confirm the correctness of the nucleotide sequence. Plasmids carrying *MCAP* coding sequence were digested with *Avr*II restriction enzyme and further transformed into either *P. pastoris* X-33 or SMD1168 using a Multiporator (Eppendorf, Hamburg, Germany), according to the supplier′s protocol. Transformants carrying *MCAP* coding sequence was identified by PCR amplification using the primers pGAP-Fw 5′-GTCCCTATTTCAATCAATTGAACAAC-3′ and AOX1-pGAP-Rv 5′-CAAATGGCATTCTGACATCCTC-3′ and by sequencing. The PCR amplification was carried out at an annealing temperature of 58 °C for 29 cycles.

### MCAP production and milk clotting activity assay

To produce MCAPs in *P. pastoris*, single colonies of positive transformants were cultured in 25 mL YPD media in a 100 mL shake flasks for 20 h at 24 °C as a starter culture and further used to inoculate 75 mL of YPD medium in 250 mL shake flasks in triplicate with a starting OD_600_ of 0.1. Cultivation was conducted for 3 days. The culture was centrifuged to obtain the crude protein extracts, later desalted using the PD-10 column equilibrated with 20 mM KH_2_PO_4_ buffer, at pH 6.0. When required, recombinant MCAP were deglycosylated by endoglycosidase H under the conditions specified by the enzyme manufacturer (New England Biolabs, Germany). Milk-clotting activity from the extracts was determined according to the method by Arima et al. (1968), with some modifications (Gama Salgado et al. 2013). Primarily, 1 mL of a substrate made of 100 g L^−1^ powder skimmed milk and 10 mM CaCl_2_ in sterile distilled water was added to a final volume of 10 mL and incubated at 35 °C for 10 min. Next, 0.1 mL of enzyme sample was added to the pre-incubated substrate. One milk clotting unit (MCU) is defined as the amount of enzyme which clotted 1 mL of the substrate in 40 min at 35 °C (Arima et al. 1968; Rao et al. 2011). Based on this definition, the milk clotting activity was calculated based on the equation of Rao and coworker (Rao et al. 2011), (Eq. 1).

1 $${\text{MCU mL}}^{ - 1} = {2 400/\left( {{\text{t }} \cdot {\text{ E}}} \right)}$$

In which 2400 is the conversion of 40 min to sec, t; clotting time (s) while E; the enzyme volume (mL).

### Purification of MCAP

All purification experiments were conducted using an ÄKTA purifier system (GE Healthcare, Munich, Germany). Upon removal of the cells debris by centrifugation at 4000*g*, 4 °C, the MCAP recombinant proteins were purified by cation-exchange chromatography utilizing a 5 mL HiTrap SP FF column mounted to the ÄKTA purifier. Protein extracts were adjusted to pH 3.1 utilizing citric acid, and then 37–48 mL of the sample mixture was injected to the previously equilibrated column (75 mM NaCl and 50 mM citric acid at pH 3.5). After washing with the same buffer, step gradient elution in 5 column volumes with a flow rate of 1 mL min^−1^ using 200 mM NaCl and 50 mM citric acid at pH 3.5 was conducted. Purified fractions were collected and analyzed for protein content and milk clotting assays.

### Deglycosylation assay

Crude extracellular protein amounting to 35 µg, previously desalted using an equilibrated (20 mM KH_2_PO_4_ buffer, pH 6.0) PD-10 column was digested with 2 units of endo H (New England Biolabs, Frankfurt, Germany) at 37 °C for 2 h.

### Protein determination

Protein content in the crude extract, supernatant broth, as well in the chromatographic fractions was determined as per Bradford procedure (Bradford 1976) and bovine serum albumin was used as a standard (Fischer Scientific, Schwerte, Germany).

### SDS-PAGE analysis

After separation of *P. pastoris* transformants culture by centrifugation, the supernatant proteins were desalted utilizing a PD-10 column (GE Healthcare, Munich, Germany) equilibrated with 20 mM KH_2_PO_4_ buffer at pH 7.2. When needed, the crude protein was concentrated at room temperature using Vacuum Concentrator 5305 (Eppendorf, Hamburg, Germany) and loaded on a 12.5% SDS–polyacrylamide gel electrophoresis. The crude authentic MCAP (control) produced by *M. circinelloides* and purified MCAP were also loaded directly on the SDS-PAGE gel for electrophoresis and was stained in Coomassie brilliant blue solution.

### Thermostability of the recombinant enzymes

The purified protein thermostability was determined by incubating the enzyme samples at temperature values between 35 °C to 65 °C for 10 min. After incubation for 10 min, samples were chilled in an ice bath prior to assay for analyzing of residual milk-clotting activity.

### Proteolytic activity assay

The obtained chromatographic fractions were further used for proteolytic activities (PA) analysis based on the method by Fan and coworkers using N, N-dimethyl casein (DCM) as the substrate (Fan et al. 2010). For the sample analysis, 10 mg of DCM was dissolved in 1 mL of 20 mM phosphate buffer, pH 5.8. Subsequently, 45 μL of enzyme sample was thoroughly mixed with 45 μL of the solution and incubated at 35 °C for 30 min. To end the reaction, 1.35 mL of 10% ice-cold trichloroacetic acid (TCA) was used and the samples were kept on ice for 30 min. The samples were later centrifuged at 15,000*g* for 15 min and the absorbance of the mixture was measured at 280 nm. For the reference solution, TCA was added before the enzyme. One unit of proteolytic activity (U mL^−1^) is defined as the quantity in microgram of tyrosine released from DCM per minute at 35 °C. The extinction of tyrosine was taken to be 0.005 mL μg^−1^ cm, (Eq. 2).

2 $${\text{PA }}\left( {\left( {\text{U/mL}} \right){ = }\left( {{\text{A}}_{{ 2 8 0 {\text{ nm}}}} / 0. 0 0 5} \right) \, \times { 1} . 4 4 {\text{ V }} \times \, \left( { 1 / 3 0} \right) \, \times { 1000/ 45}} \right)$$

where V is a volume in mL.

## Results

### Sequence analysis of the Signal peptide of MCAP (mcSP)

**Fig. 1:**
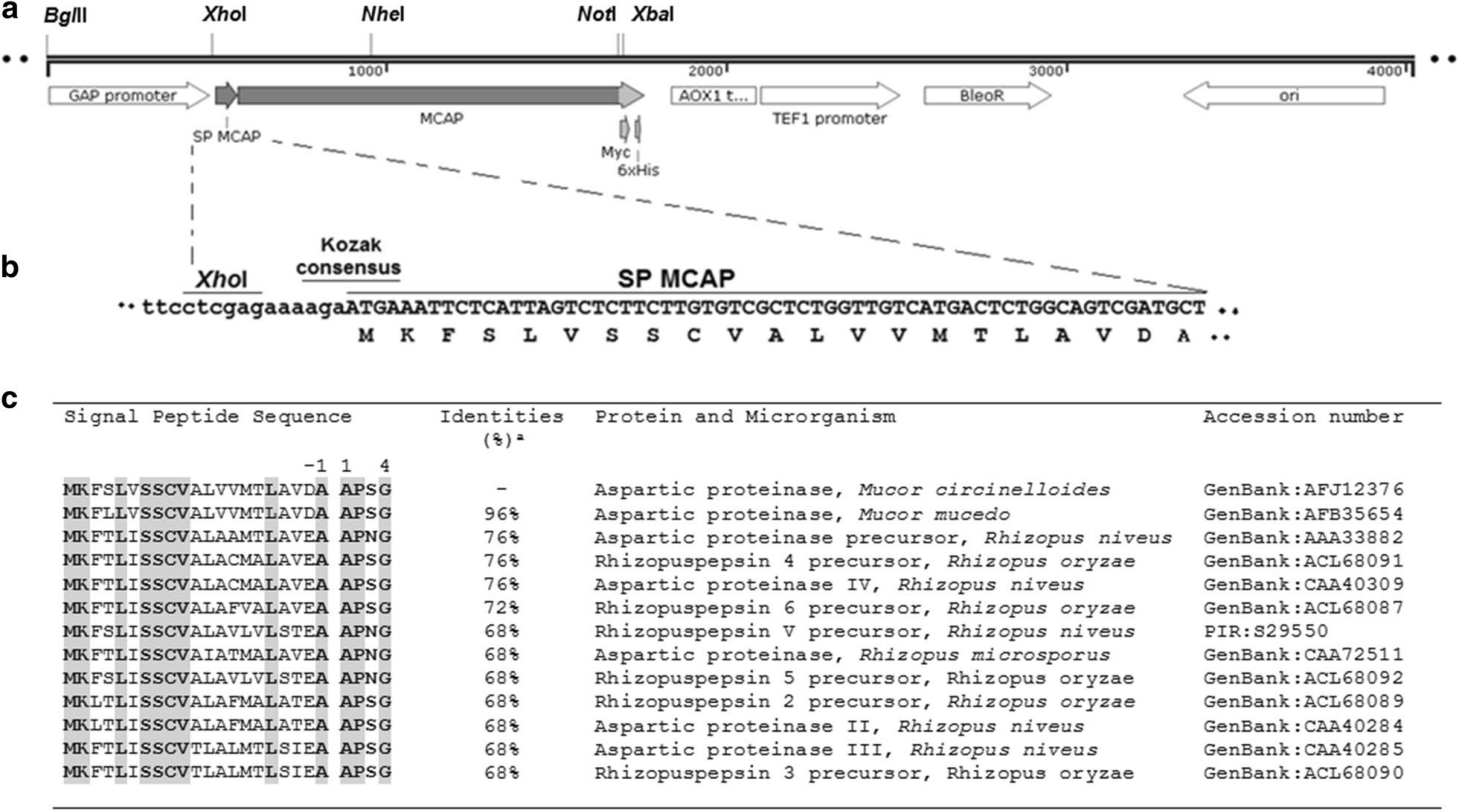
Recombinant plasmid construction pGAM1 + MCAP-3: **a** map of pGAM1 + MCAP-3, **b** diagram of the *MCAP* gene showing the Kozak consensus located between the GAP promoter and the SP MCAP sequence of pGAM1 + MCAP-3. **c** The identity of the signal peptide sequence (SP MCAP) of *M. circinelloides* with other fungal counterparts. The residues are numbered according to the signal sequence cleavage (between position − 1 and 1: A-AP). The residues identical are shaded in grey

Most proteins that are secreted by organisms possess a signal peptide sequence, which is cleaved by signal peptidase (SPase) upon maturation (Blobel 2000). In particular, all the fungal aspartic proteases sequences reported so far have a signal peptide. In our studies, the gene that encodes the MCAP protein was PCR amplified from a fungal plant pathogen strain *M. circinelloides* DSM 2183 and a 1229 bp including one intron of 63 bp long fragment (Fig. 1a, b, Additional file 1: Table S1) was obtained. It consists of a signal peptide (SP) and the fragment showed the presence of catalytic Asp residues commonly found in most known aspartic proteinases. The catalytic Asp residues are usually found within the motif Asp–Xaa–Gly where Xaa can either be Ser or Thr (Rao et al. 2011). Additionally, the SP was predicted using the SignalP 4.0 server (Center for Biological Sequence Analysis, Technical University of Denmark, DTU) that recognized a cleavage signal sequence site to be between the amino acid positions Ala-21 and Ala-22 (Fig. 1c). According to the deduced amino acid sequence, MCAP has a putative pre-pro region of 72 amino acid (aa) residues, preceded by the mature enzyme of 322 aa. The *M. circinelloides* signal peptide (mcSP) corresponding to the first 21 aa (MKFSLVSSCVALVVMTLAVDA) shows typical features for targeting a protein to the membrane, such as a highly hydrophobic region and a pro-enzyme sequence consisting of about 51 aa (Gama Salgado et al. 2013).

In this study protein secretion using the native mcSP was analyzed and it was found that the MCAP was secreted into the culture medium at a concentration 200 mg L^−1^ while MCAP with an α-MF was at 110 mg L^−1^, respectively, after three days of cultivation. From the data obtained, it was observed that the strain containing the *MCAP* cloned downstream of the α-factor signal sequence produced an enzyme with 249 U (Milk clotting units mL^−1^), while recombinants X-33/pGAM1 + MCAP-3 and SMD116/pGAM1 + MCAP-3 carrying the mcSP expressed 410 U and 415 U, respectively. The MCAP protein produced by the recombinant yeast (X-33/pGAM1 + MCAP-3 and SMD116/pGAM1 + MCAP-3) was estimated to be 200 mg L^−1^ and Table 2 summarizes the expression levels between mcSP and α-factor signal.

**Table 2:** Expression and activity analysis between mcSP-MCAP and α-MF-MCAP

Strain	Signal peptide	Crude extract (mg L^−1^)	Milk clotting (units mL^−1^)
X-33/Wild-type	–	0	0
X-33/MCAP3	α-MF + mcSP	0	0
X-33/MCAP-5	α-MF	110	249
X-33/MCAP-3	mcSP	200	410
SMD1168/MCAP-3	mcSP	200	415

Date are given as average, n = 3

The lower amount of protein with the α-MF fused could be due to the difference in the processing level of the α-MF and mcSP prepropeptides, or different levels of gene expression and stability, of which a further study in which fusing different protein varieties will be thoroughly implemented. The obtained results indicate that the mcSP is effective for protein secretion in yeast cells and can be used as an alternative to the alpha-factor SP mainly used in yeast protein secretion.

### Protein expression and analysis of the N-glycosylation sites by site-directed mutagenesis

**Fig. 2:**
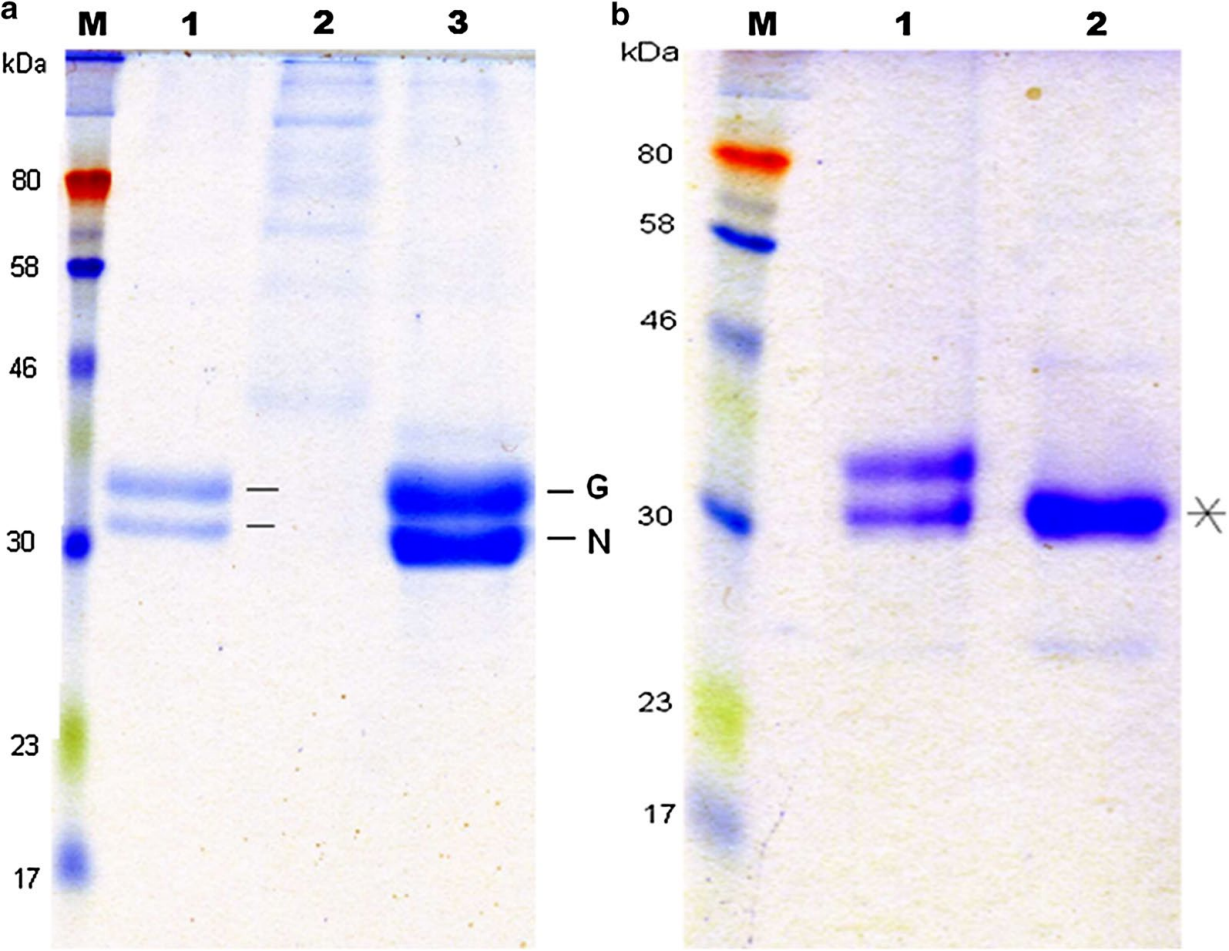
SDS-PAGE analysis of the extracellular extract from recombinants; **a** lane 1 X-33/pGAPZα + MCAP-5, lane 2 X-33/wildtype, lane 3 X-33/pGAM1 + MCAP-3. **b** Enzymatic analysis of the extracellular extract from X-33/pGAM1 + MCAP-3 with endoglycosidase (Endo H) lane 2

Previously it was reported that *P. pastoris*, carrying the plasmid pGAPZαA + MCAP-5 produced two forms of extracellular MCAP: glycosylated and non-glycosylated and a further digestion of the MCAP with endoglycosidase H caused a decrease in the superficial molecular weight (Gama Salgado et al. 2013). Therefore, yeast strain X-33/pGAPZα + MCAP-5, X-33/wildtype, X-33/pGAM1 + MCAP-3 were cultured in YPD medium consisting of 4% glucose at 24 °C for 3 days at pH 5.0 and Fig. 2a shows an SDS-PAGE gel of the extracellular extract with arrows indicating the expressed types of MCAP protein (G and N, Glycosylated and Non-glycosylated, respectively). Furthermore, enzymatic analysis of the extracellular extract from X-33/pGAM1 + MCAP-3 with endoglycosidase (Endo H) showed a decrease in the molecular weight (33 kDa) indicating that the protein was initially expressed in the glycosylated form (Fig. 2b, lane 2).

**Fig. 3:**
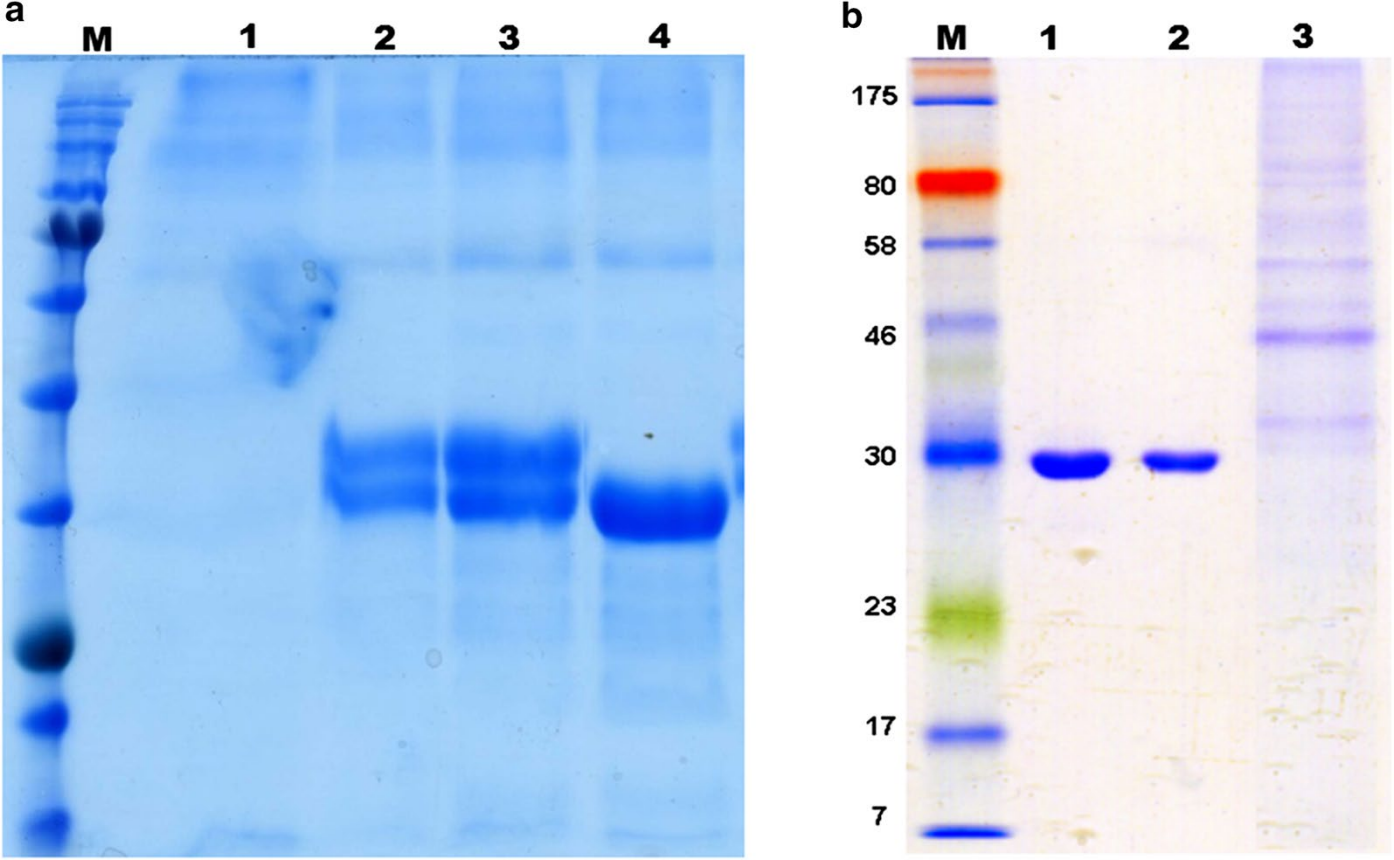
SDS-PAGE analysis of the extracellular extract from recombinants; **a** lane 1 X-33/wild-type, lane 2 X-33/pGAPZα + MCAP-5, lane 3 X-33/pGAM1 + MCAP-3, lane 4 X-33/pGAM1 + MCAP-8, and **b** purified MCAP-3 digested with endoglycosidase (Endo H) lane 1 and purified MutMCAP-8 in lane 2

By using NetNGlyc v1.0. Software, one potential N-glycosylation site (Asn–X–Ser/Thr) was found to be located at positions Asn331 and any possible O-glycosylation sites were also predicted by NetOGlyc v3.1. Therefore, to show and proof the presence of N-glycosylation sites on aspartic proteinase of *M. circinelloides* produced in *P*. *pastoris*, a mutant where N (Asn331) was replaced with Q (Gln) was created by site-directed mutagenesis and was successfully expressed in *P*. *pastoris*. The cultivation of the mutant and non–mutants (Table 3) was carried as described in “Materials and methods” section. After cultivation, the cells were separated from the medium and the supernatants were analyzed by SDS-PAGE. From the SDS-PAGE analysis (Fig. 3a), it can be seen that the recombinants in lane 4 expressed a single MCAP protein having a molecular weight of almost 33 kDa compared to lane 2 and 3, where two bands where observed. From these results, we can conclude that the protein was glycosylated at the Asn331 position. The mutant protein Asn331-Gln and the wild-type protein were further deglycosylated with Endo H. We can see from Fig. 3b lane 1 and 2 that upon deglycosylation, the protein molecular weight of almost 33 kDa was seen for both, a band of the molecular weight similar that of line 4 in Fig. 3a. From these results, we can confirm that native MCAP is produced in both unglycosylated and glycosylated form.

**Table 3:** Summary of production and purification of MCAP

Sample	Step	Volume (mL)	Protein^a^ (mg)	Recovery (%)	Purification (fold)
MCAP-3 (non-mutated)	Crude	49	8.44	100	1
	IEX	12.5	4.67	55.3	2.1
SyMCAP-6 (synthetic)	Crude	50	14	100	1
	IEX	12.5	9.17	65.5	1.5
MutMCAP-8 (mutated)	Crude	48	5.76	100	1
	IEX	7.5	3.5	60.7	6.6

^a^Date are given as average, n = 3

### Optimum pH

**Fig. 4:**
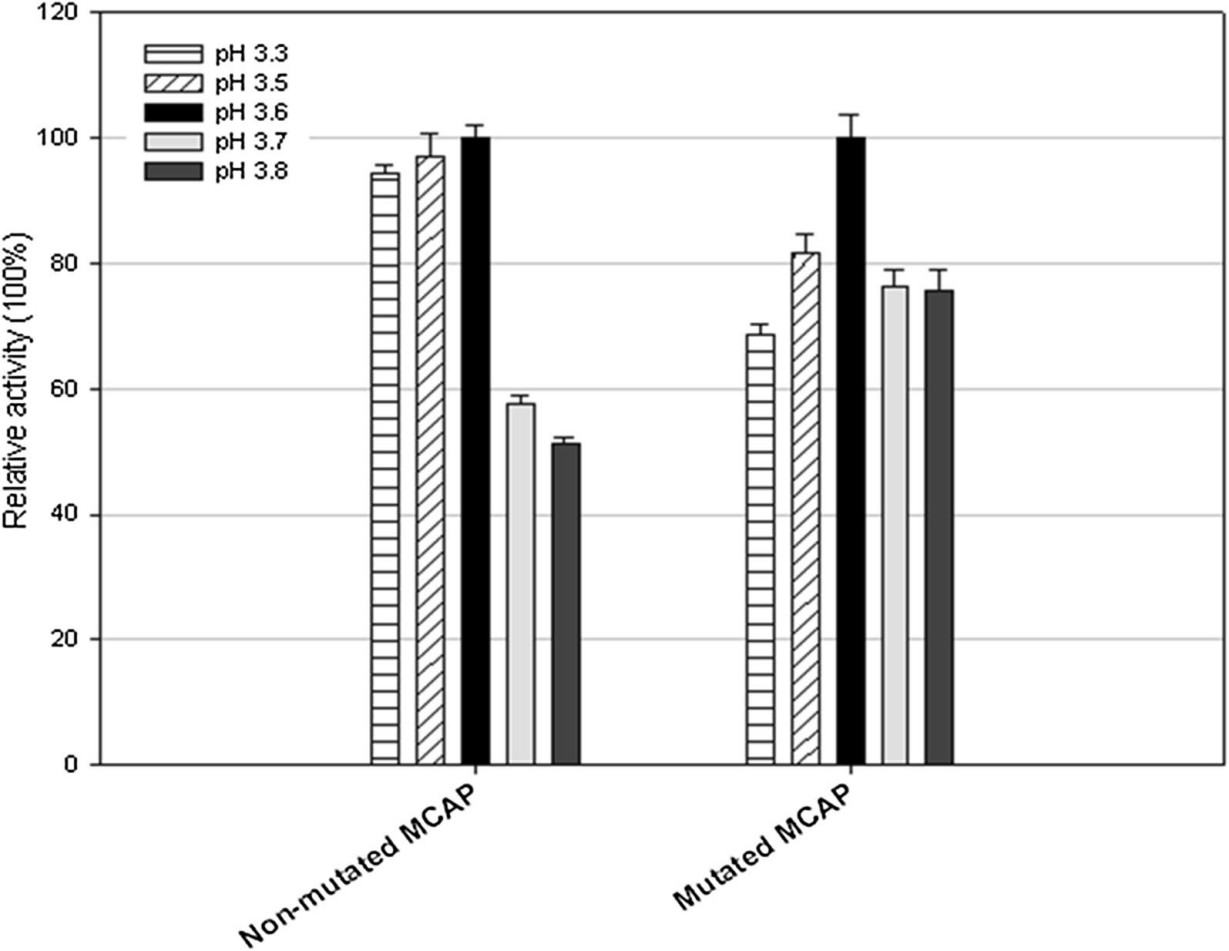
Effect of pH on MCAP activity

To further analyze the pH stability of the MCAP, the enzyme preparations were initially incubated at 30 °C for 1 h in 20 mM citric acid buffers within a pH range of 3.3 and 3.8. Figure 4 shows that the maximum enzyme activity for both mutated and non-mutated was at pH 3.6. Previous results showed that MCAP enzyme was stable between pH 3.0 and 4.0, though at a pH of 7.0 the activity decreased drastically. Fernandez- Lahore et al. reported a similar behaviour of an acid proteinase from mesophilic *Mucor* sp. were it was also found that MCAP enzyme is not stable under the same conditions above a pH of 7.5 (Fernandez-Lahore et al. 1999).

### Optimum temperature and thermostability of MCAP enzymes

**Fig. 5:**
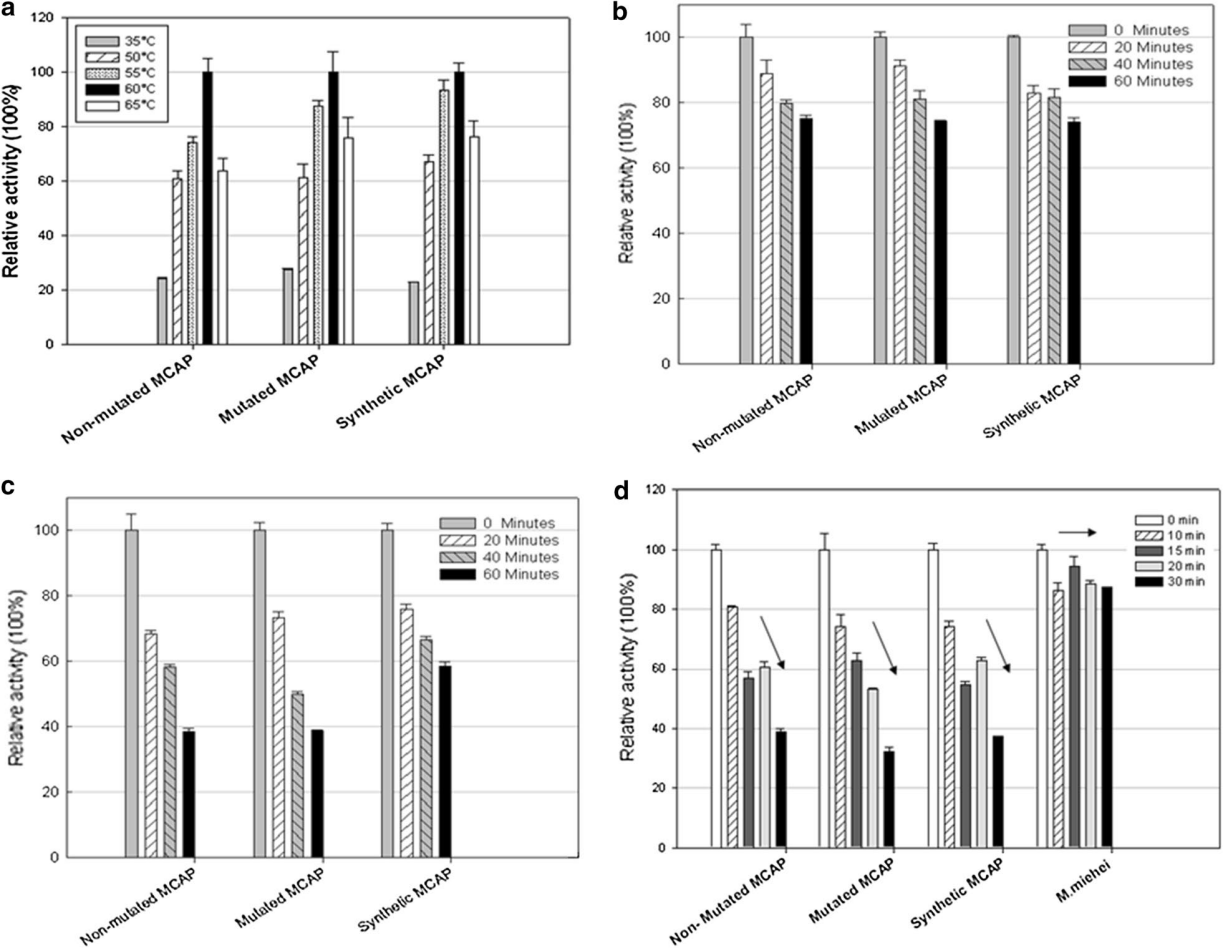
Effect of; **a** Optimum temperature of MCAP, **b** Thermostability of the unpurified MCAP at 55 °C, **c** Thermostability of the unpurified MCAP at 60 °C, **d** Thermostability of the purified MCAP at 55 °C

The MCAP enzyme activity was determined as a function of temperature from 35 to 65 °C and Fig. 5a shows that the highest enzyme activity was at 60 °C irrespective of the type of protein. In specific instances, upon 60 min incubation period enzyme activity begins to decrease at a temperature above of 50 °C. When unpurified MCAP′s where tested, it was found that after 60 min of incubation at 55 °C, 75% of enzyme activity was retained (Fig. 5b) and 40–60% of the enzyme activity at 60 °C (Fig. 5c) upon a 60 min incubation period at pH 3.6. As indicated in Fig. 5d, the retained activity of the purified MCAP varies considerably to the non-purified protein. Results also showed that there was less or no effect of glycosylation on the thermostability of the purified enzymes (Fig. 5d). From the results, it can be noted that a difference exists between crude and the purified samples on the point of thermal stability. This might be due to other elements in the culture medium that might affect protein integrity. Though, from the SDS-PAGE pattern of the crude enzyme samples obtained in this study (Fig. 3), we can clearly see that the aspartic protease produced in *P*. *pastoris* is the main protein in the culture supernatant. Generally, enzyme thermostability is an inherent property, determined mainly by the protein primary structure. However, external factors like cations, substrates, co-enzymes, modulators and other proteins often affect enzyme thermostability.

The vicinity of N-glycosylation site to the catalytic site may also have a potential effect on thermal and biochemical parameters of the enzyme, therefore possible effects should be studied by a further detailed structural analysis.

### Proteolytic activity

Understanding proteolytic and milk clotting activity ratio is a vital criterion for the selection of a good milk coagulant, as it has been observed that the higher the ratio the more desirable the order of the action during cheese making. In this study, the proteolytic activity of the purified protein was determined at pH 5.8, at 35 °C. The differences in proteolytic activity were recorded after 30 min of incubation (*N*,*N*-dimethyl casein was used as the substrate). Table 4 shows the Milk-clotting activity (MCA), Proteolytic activity (PA) and the MCAP/PA ratio. This result indicates that glycosylation may have an effect on the milk clotting and proteolytic activity. Although the mutated protein showed the highest proteolytic activity no differences in the MCAP/PA ratio were observed in relation to the non-mutated protein. From the results, we can say that nonglycosylated enzymes could also be a good alternative to the existing preparations for cheese manufacturing.

**Table 4:** Milk clotting and proteolytic activity of purified MCAP and its mutants

Sample	MCA (U µg^−1^)	Proteolytic activity PA (U µg^−1^)	Ratio MCA/PA
MCAP-3 (non-mutated)	72	4.26 ± 0.82	17.35 ± 3.75
SyMCAP-6 (synthetic)	137	7.02 ± 0.28	19.53 ± 0.79
MutMCAP-8 (mutated)	204	13.56 ± 1.47	15.13 ± 1.64
*M. mihei*	311	11.11 ± 0.27	28 ± 0.68^a^

Results shown are the means of three sets of experiments

^a^Data from Gama Salgado et al. (2013)

## Discussion

To investigate the expression of *MCAP* gene in *P. pastoris*, several plasmid constructions harbouring intact or partial *MCAP* gene sequence were generated. Initially, a novel *M. circinelloides* aspartic protease gene (*MCAP*) was cloned into pGAPZαA, a commercial plasmid (Invitrogen, Darmstadt, Germany) that expresses protein using a constitutive *GAP* promoter with a secretion signal sequence (the S. cerevisiae α-MF pre-pro signal sequence) for secreting needed protein in the culture media (Gama Salgado et al. 2013). No MCAP was secreted when MCAP was produced without the prepropeptide sequence, peptide important for correct folding during secretion in zymogens (Mellor et al. 1983; *Gray* et al. 1986; Tonouchi et al. 1986; Horiuchi et al. 1988; Hiramatsu et al. 1989), nor when the secretion signal (α-MF) of *S. cerevisiae* (Brake et al. 1984) followed by mcSP was used in the expression system (Table 2). This phenomenon can be called incompatibility of SP sequence. Furthermore, the strain carrying the intact *MCAP* gene (with intron sequence) was not able to produce MCAP. This might be due to *P. pastoris* inability to cleave the *MCAP* gene intron sequence, as it was also observed by Horiuchi et al. in *S. cerevisiae* when the *R. niveus* aspartic proteinase containing an intron sequence was used (Horiuchi et al. 1988). On the other hand, when the intron of the *MCAP* gene was removed, the recombinant strain secreted MCAP into the medium at a concentration exceeding 110 mg L^−1^ (249 MCU mL^−1^) a level not comparable to the MCAP expressed in the fungus *M. circinelloides* (800–1000 MCU mL^−1^). For efficient secretion of MCAP in *P. pastoris*, the *MCAP* gene sub-cloned into a new expression vector pGAM1 (pGAM1 without the *S. cerevisiae* α-MF pre-pro signal sequence) and the level of protein expression between mcSP and α-MF was compared. When using the mcSP, the aspartic proteinase was secreted in active form into the culture medium at a concentration exceeding 250 mg L^−1^ (1000 MCU mL^−1^) indicating a 1.5-fold increase in the secretion of MCAP by *P. pastoris* as compared to when the α-MF secretion signal of *S. cerevisiae* was used. The reason for increased secretion could be that the processing of α-MF require three steps; removal of peptide signal, *Kex2* endopeptidases cleavage and repeats of the Glu-Ala cleavage), while the processing of mcSP need only one, the removal of the peptide signal and this provides an advantage over the existing commercial expression vector. Furthermore, the number of genes in *P. pastoris* were analyzed by the real‐time PCR‐based method and no differences were observed among the strains.

Additionally, when the original *MCAP* gene was adapted to the optimal codon usage of *P. pastoris,* the production of heterologous MCAP was increased three folds. The synthetic gene is 78% identical (nucleotide sequence level) to the original and the observed increase in production of several other recombinant proteins in *P. pastoris* using codon optimization has also been reported in the literature (Sinclair and Choy 2002; Xiong et al. 2005; Gao et al. 2013).

This study also shows that MCAP is N-glycosylated at Asn331 when produced in *P. pastoris*, and no much difference was observed on the effect of the N-glycosylation on the stability and activity of the protein, as the proteolytic (PA) and milk clotting activity (MCA) ratios were similar between MCAP-3 and mutMCAP-8 (Table 4). Rao et al. (2011) reported that most aspartic proteinases show maximal activity between pH 3 and 4, and their molecular masses range from 30 to 45 kDa, though some can be higher.

With the data obtained, non-glycosylated protein can also be used as a better candidate for cheese production. Literature data have shown that the sequon Asn-Xaa-Thr rather than Asn-Xaa-Ser (Daly and Hearn 2005) is readily glycosylation as we have already observed in the data obtained in this study. The position of sequon in relation to the protein C-terminus was also labelled as a decisive factor for extended glycosylation since the sequence of the consecutive amino acids which are situated close to the N-terminus have been noted to be prone to glycosylation than those closer to the C-terminus (Shelikoff et al. 1996). Experimental results by Gavel and Gunnar von Heijne (1990) also indicate that the N-glycosylation site does not always lead to glycosylation, especially when the sites are at the C-termini of proteins. Remarkably, the MCAP comprises the consecutive amino acids situated near the C-terminus (Asn331 in the MCAP of 394 amino acid residues). Therefore, *P. pastoris* is capable of excreting two types of MCAPs. Additional data obtained by Shakin-Eshleman et al. (Mellquist et al. 1998), propose that a specific amino acid at the *X* location of an Asn-*X*-Ser sequon is vital for *N*-linked Core-Glycosylation Efficiency (CGE). It was observed that the replacement of the amino acid X with Phe, improved the efficiency of core glycosylation (mean CGE = 70%) and in our case, the protein contains Phenylalanine at the X position of the sequon. The results indicate that the intensity of the band showing glycosylated recombinant protein was more intense than the recombinant non-glycosylated one.

Also, we observed that glycosylation had no significant effect on the thermostability of the purified protein, as both glycosylated and unglycosylated proteins revealing similar clotting activity and proteolytic activity ratios. In this study, 83% of the residual activity of commercial rennet from *R*. *miehei* after exposure to heat for 30 min was observed (Fig. 5d), while that of purified MCAP was less than 40%. The fact that the protein has low thermal stability and relatively high milk clotting activity to proteolytic activity ratio, the enzyme(s) can be a better candidate for commercial rennet preparations for the cheese industry.

The *P*. *pastoris* expression system used in this study resulting in functional secretion of the *M*. *circinelloides* aspartic proteinase and its mutant forms can further be used in the production of the enzyme in large scale.

It was also found that the native signal peptide (mcSP) was effective for protein secretion in yeast cells and could be used as an alternative to the alpha-factor SP mainly used in yeast protein secretion. Available data (von Heijne 1983) has shown that sequences leaders possess markedly three regions (n-region, h-region and c-region), which consists of specific amino acids that make up the primary structure of the protein. The n-region varies from one to five amino acids in length from the initiator methionine to the region-h and has a positive charge, while the region-h is a central hydrophobic core varies from 8 to 12 residues (Nielsen and Krogh 1998; Laforet and Kendall 1991). In relation to the region c, it is known that this region is polar containing 3 to 7 amino acids but most of the residues are not charged. It should be noted that the region c is involved in the recognition and cleavage by signal peptidase. Particularly, the amino acids at the location -3 and -1 in relation to the cleavage site, plays an important role in the correct signal peptide cleavage (von Heijne 1983). Furthermore, it has been shown that the characteristics of the mature amino terminus should be involved in determining the exact cleavage site (Laforet and Kendall 1991). Taking this prediction, the mcSP sequence was searched against the GenBank database for non-redundant protein using the BLASTP 2.2.27 program. Interestingly, the results obtained indicated that mcSP has several amino acid residues present in aspartic proteinases produced by various fungi (Fig. 1). We also found that the first four amino acids from the N-terminal sequence of aspartic proteinases were conserved in at least three residues. In addition, we found that some of the N-terminal residues of mature MCAP at positions +1 to +5 to the relative cleave site of mcSP are present in the same position of a secreted protein native to *P. pastoris*. Based on this result, we analyzed the α-MF of *S. cerevisiae* including some heterologous signal peptides, which have shown a proper functioning in *P. pastoris*. As an illustration, we compared these sequences including the first 10 amino acids just after the cleavage site to 54 native signal peptide sequences of *P. pastoris,* which were recently predicted (De Schutter et al. 2009), and some residues of the α-MF pro-sequence (Waters et al. 1988; He et al. 2012) matched with N-terminal amino acids of a mature protein of *P. pastoris*. In addition, we observed similar results with the glucoamylase (Chen et al. 2007), pectate lyase B (Guo et al. 1995), laccase (Liu et al. 2003), alpha-1-antitrypsin (Arjmand et al. 2013), urine protein complex (Ferrari et al. 1997), acetylcholinesterase (Morel and Massoulie 1997) and alpha amylase signal peptide (Li et al. 2011).

In a comparative analysis, only part of the secretome predicted to *P. pastoris* was used (De Schutter et al. 2009). Possibly some of the sequences analyzed might match better with other signal peptides of the yeast. These results presented here can perhaps contribute to the improvement of heterologous protein secretion.

This study provides guidance for the enhanced enzyme production focusing on the utilizing the native SP and also the N-linked glycosylated modification that can help meet the imminent needs of industries and can offer an excellent biocatalyst for the application in other biotechnological industries.

Our next tasks will be to quantitatively analyze gene expression and evaluate the effectiveness of utilizing mcSP in various protein secretion.

## Additional file

**Additional file 1: Table S1.** The Nucleotide and deduced the amino acid sequence of MCAP protein. The deduced amino acid sequence is shown under the nucleotide sequence. The arrow indicates the signal peptide cleavage site and lowercase letters indicate nucleotides in the intron sequence. The catalytic Asp residues (motifs DTGS and DTGT) are boxed. The N-glycosylation site is single underlined. Asterisk indicates the position of the stop codon (TAA).

## Abbreviations

MCAP: *Mucor circinelloides* aspartic protease
mcSP-MCAP: signal peptide *Mucor circinelloides* aspartic protease
MCU: milk clotting units
