# Supplementary material for: Identification and characterization of N-glycosylation site on a Mucor circinelloides aspartic protease expressed in Pichia pastoris: effect on secretion, activity and thermo-stability
Source: AMB Express. 2018 Oct 1;8:157. doi: 10.1186/s13568-018-0691-3 (PMC6167268; doi:10.1186/s13568-018-0691-3)
Supplement: Supplementary file 1 — Additional file 1: Table S1. The Nucleotide and deduced the amino acid sequence of MCAP protein. The deduced amino acid sequence is shown under the nucleotide sequence. The arrow indicates the signal peptide cleavage site and lowercase letters indicate nucleotides in the intron sequence. The catalytic Asp residues (motifs DTGS and DTGT) are boxed. The N-glycosylation site is single underlined. Asterisk indicates the position of the stop codon (TAA). [file 13568_2018_691_MOESM1_ESM.doc]

Additional file 1: Table S1. The Nucleotide and deduced amino acid sequence of MCAP protein

| 1 1 103 35 205 69 307 103 409 116 511 150 613 184 715 218 817 251 919 286 1021 320 1123 354 1225 388 | ATGAAATTCTCATTA GTCTCTTCTTGTGTC GCTCTGGTTGTCATG ACTCTGGCAGTCGAT GCTGCTCCCAGTGGT AGCAAGAAGCTTTCC GTTCCTTTGGCT  M  K  F  S  L   V  S  S  C  V   A  L  V  V  M   T  L  A  V  D   A  A  P  S  G   S  K  K  L  S   V  P  L  A  AAAAATGAAGACTAC CAACCCAACATCAAG CGCTCCATTGCTAAA GCCCGTGCAAAGTAT ATCAAGCACATTATC AACCCCCTCAAGGGT GTTCCCGCCGGC  K  N  E  D  Y   Q  P  N  I  K   R  S  I  A  K   A  R  A  K  Y   I  K  H  I  I   N  P  L  K  G   V  P  A  G  GCTACTACTGATGCC ACTGGTACTGTCCCC GTTACTGATTACGCA AACGACATTGAATAC TATGGTACTGTCAAG GTCGGTACTCCCGCT CAATCCCTCAAG  A  T  T  D  A   T  G  T  V  P   V  T  D  Y  A   N  D  I  E  Y   Y  G  T  V  K   V  G  T  P  A   Q  S  L  K  ATCAACTTTGATACT GGTTCCTCTGATTTC TGGTTTG**gtaagact aatgatgtacatatt tatgtgtagagactt cattatctaacctca attataatag**CT  I  N  F  **D  T   G  S**  S  D  F   W  F   A  TCCACTTTGTGCTCT ACTTGTACCACTCAC ACTCGTTATGATCCC ACCAAGTCCAGCACC TATGTTGCTGATGGT CGTGCCTGGTCTATC CAATACGGTGAT  S  T  L  C  S   T  C  T  T  H   T  R  Y  D  P   T  K  S  S  T   Y  V  A  D  G   R  A  W  S  I   Q  Y  G  D  GGTTCCACTGCTAGC GGTGTCTTGGCCAAG GATACTGTCAACTTG GGTGGTCTCACCATC AAGAGCCAAACTATC AACTTGGCCAAGAAG GAATCCAGCAGT  G  S  T  A  S   G  V  L  A  K   D  T  V  N  L   G  G  L  T  I   K  S  Q  T  I   N  L  A  K  K   E  S  S  S  TTTGCTAGTGATCCT ATTGATGGTCTTATG GGTCTCGGTTTTGAC ACCATCACCACTGTC GCTGGTATCAAGACT CCTGTTGATAACTTG ATCAGCCAAGGT  F  A  S  D  P   I  D  G  L  M   G  L  G  F  D   T  I  T  T  V   A  G  I  K  T   P  V  D  N  L   I  S  Q  G  TTGATCTCTTCTCCT GTTTACGGTGTCTGG CTCGGTAAGGCCAAG AATGGTGGTGGTGGT GAATACTTGTTTGGT GGTTCCAACCCCAAC CATTACACTGGT  L  I  S  S  P   V  Y  G  V  W   L  G  K  A  K   N  G  G  G  G   E  Y  L  F  G   G  S  N  P  N   H  Y  T  G  GCTTTGACTACTGTT CCTGTCGATAACTCT CAAGGCTTCTGGGGT ATTACAGTTGGTAGT CTCAAGGCTGGTACT ACCTCTGTCACTGGT TCTTTCAGCGGT  A  L  T  T  V   P  V  D  N  S   Q  G  F  W  G   I  T  V  G  S   L  K  A  G  T   T  S  V  T  G   S  F  S  G  ATCCTTGATACTGGT ACCACTCTCTTGCTC TTCCCCCAATCCATT GCCAACAAGGTTGCT GCTCAATACGGTGCC AGAGACAATGGTGAC GGTACTTACACT  I  L  **D  T  G   T**  T  L  L  L   F  P  Q  S  I   A  N  K  V  A   A  Q  Y  G  A   R  D  N  G  D   G  T  Y  T  ATCAGCTGTAGCACC GCCAACCTCAAGCCT CTTAACTTCACCATC AACGGTGCTCAATTC CAAGTTCCTGTTGAT TCCCTCATCTTTGAA CAGGATGGTTCC  I  S  C  S  T   A  N  L  K  P   L  **N  F  T**  I   N  G  A  Q  F   Q  V  P  V  D   S  L  I  F  E   Q  D  G  S  ACTTGTTATGCAAGC TTCGGTTACGCTGGC CTTGACTTTGCTATT TTGGGTGATGTCTTC TTGAAGAACAACTAT GTTATCTTTAACCAA AAGGTTCCTCAA  T  C  Y  A  S   F  G  Y  A  G   L  D  F  A  I   L  G  D  V  F   L  K  N  N  Y   V  I  F  N  Q   K  V  P  Q  GTCCAAATTGCCAAA TCTGTCTAA  V  Q  I  A  K   S  V  ***** |
| --- | --- |

The deduced amino acid sequence is shown under the nucleotide sequence. The arrow indicates the signal peptide cleavage site and lowercase letters indicate nucleotides in the intron sequence. The catalytic Asp residues (motifs DTGS and DTGT) are boxed. The N-glycosylation site is single underlined. Asterisk indicates the position of the stop codon (TAA)
